# Supplementary material for: How to Use Lean Thinking for the Optimization of Clinical Pathways: A Systematic Review and a Proposed Framework to Analyze Pathways on a System Level
Source: Healthcare (Basel). 2023 Sep 7;11(18):2488. doi: 10.3390/healthcare11182488 (PMC10530850; doi:10.3390/healthcare11182488)
Supplement: Supplementary file 1 [file healthcare-11-02488-s001.zip › healthcare-2532531-supplementary.pdf]

Table S1. Full data set with included articles and collected outcomes

Included article	Year of publication	Lean or Lear	Continuous improvement elements	4 P model	Continuous improvement methodoloy	Type of outcome
Aasebo U, Strom HH, Postmyr M. The Lean method as a clinical pathway facilitator in patients with lung cancer. Clin Respir J 2012;6(3):169-74. doi: 10.1111/j.1752-699X.2011.00271.x	2012	Lean	VSM<br>Standardisation of Work	Process<br>Process	Not described	Process
Boafo A, Greenham S, Cloutier P, et al. Development of a Clinical Pathway for the Assessment and Management of Suicidality on a Pediatric Psychiatric Inpatient Unit. Adolesc Health Med Ther 2020;11:123-33. doi: 10.2147/AHMT.S240060	2020	Lean	A3<br>Process mapping<br>Standardisation of Work	Problem Solving<br>Process<br>Process	Not described	NA
Bradywood A, Farrokhi F, Williams B, et al. Reduction of Inpatient Hospital Length of Stay in Lumbar Fusion Patients With Implementation of an Evidence-Based Clinical Care Pathway. Spine (Phila Pa 1976) 2017;42(3):169-76. doi: 10.1097/BRS.0000000000001703	2017	Lean	VSM<br>5 Whys<br>Cause-and-Effect Diagram	Process<br>Problem Solving<br>Problem Solving	Not described	Process<br>Quality<br>PREMs
Ciulla TA, Tatikonda MV, ElMaraghi YA, et al. Lean Six Sigma Techniques to Improve Ophthalmology Clinic Efficiency. Retina 2018;38(9):1688-98. doi: 10.1097/IAE.0000000000001761	2018	LSS	Cause-and-Effect Diagram<br>Process mapping<br>Continuous Flow	Problem Solving<br>Process<br>Process	DMAIC	Process
Didden AGM, Punt IM, Feczko PZ, et al. Enhanced recovery in usual health care improves functional recovery after total knee arthroplasty. Int J Orthop Trauma Nurs 2019;34:9-15. doi: 10.1016/j.ijotn.2019.03.003	2019	LSS		Process	DMAIC	Clinical<br>Process<br>Quality
Frangeskou M, Lewis MA, Vasilakis C. Implementing standardised flow: navigating operational and professional dependencies. Int J Oper Prod Manage 2020;40(7/8):1177-99. doi: 10.1108/ijopm-06-2019-0493	2020	Lean	Continuous Flow	Process	Not described	NA (qualitative outcomes)
Funderburk CD, Batulis NS, Zelones JT, et al. Innovations in the Plastic Surgery Care Pathway: Using Telemedicine for Clinical Efficiency and Patient Satisfaction. Plast Reconstr Surg 2019;144(2):507-16. doi: 10.1097/PRS.0000000000005884	2019	LSS	Process mapping	Process	PDCA	PREMs
Gayed B, Black S, Daggy J, et al. Redesigning a joint replacement program using Lean Six Sigma in a Veterans Affairs hospital. JAMA Surg 2013;148(11):1050-6. doi: 10.1001/jamasurg.2013.3598	2013	LSS	Single-piece flow<br>VSM<br>Waste reduction	Process<br>Process<br>Process	Not described	Process<br>Cost
Goga JK, Michaels A, Zisselman M, et al. Reducing opioid use for chronic pain in older adults. Am J Health Syst Pharm 2019;76(8):554-59. doi: 10.1093/ajhp/zxz025	2019	Lean	A3<br>Process mapping<br>Waste reduction	Problem Solving<br>Process<br>Process	PDCA	Clinical
Haddow JB, Walshe M, Aggarwal D, et al. Improving the diagnostic stage of the suspected colorectal cancer pathway: A quality improvement project. Healthc (Amst) 2016;4(3):225-34. doi: 10.1016/j.hjdsi.2015.09.004	2016	Lean	VSM	Process	PDCA	Process
Hultman CS, Kim S, Lee CN, et al. Implementation and Analysis of a Lean Six Sigma Program in Microsurgery to Improve Operative Throughput in Perforator Flap Breast Reconstruction. Ann Plast Surg 2016;76 Suppl 4:S352-6. doi: 10.1097/SAP.0000000000000786	2016	LSS	VSM	Process	DMAIC	Process<br>Quality<br>Cost
Hydes T, Hansi N, Trebble TM. Lean thinking transformation of the unsedated upper gastrointestinal endoscopy pathway improves efficiency and is associated with high levels of patient satisfaction. BMJ Qual Saf 2012;21(1):63-9. doi: 10.1136/bmjqs-2011-000173	2012	Lean	VSM	Process	Not described	Process<br>PREMs
Improta G, Balato G, Romano M, et al. Lean Six Sigma: a new approach to the management of patients undergoing prosthetic hip replacement surgery. J Eval Clin Pract 2015;21(4):662-72. doi: 10.1111/jep.12361	2015	LSS	SIPOC<br>VSM<br>Cause-and-Effect Diagram	Process<br>Process<br>Problem Solving	DMAIC	Process
Kullar P, Harris F, Lloyd SK, et al. The use of Lean Thinking techniques in implementing the Department of Health, UK, 18-week waiting time directive for cochlear implantation. Cochlear Implants Int 2010;11(3):133-45. doi: 10.1002/cii.418	2010	Lean	VSM<br>Waste reduction	Process<br>Process	Not described	Process
Laberge M, Cote A, Ruiz A. Clinical pathway efficiency for elective joint replacement surgeries: a case study. J Health Organ Manag 2019;33(3):323-38. doi: 10.1108/JHOM-03-2018-0087	2019	Lean	VSM	Process	Not described	Process
Latessa I, Fiorillo A, Picone I, et al. Implementing fast track surgery in hip and knee arthroplasty using the lean Six Sigma methodology. The TQM Journal 2021;33(7):131-47. doi: 10.1108/tqm-12-2020-0308	2021	LSS	VSM<br>Cause-and-Effect Diagram	Process<br>Problem Solving	DMAIC	Process
Lindholm JM, Laine I, Hippala H, et al. Improving eye care services with a lean approach. Acta Ophthalmol 2018;96(7):724-28. doi: 10.1111/aos.13703	2018	Lean	VSM	Process	Not described	Process<br>PREMs
Martens L, Goode G, Wold JF, et al. Structured syncope care pathways based on lean six sigma methodology optimises resource use with shorter time to diagnosis and increased diagnostic yield. PLoS One 2014;9(6):e100208. doi: 10.1371/journal.pone.0100208	2014	LSS	Process mapping	Process	DMAIC	Process<br>Quality
Martin BD, Pestieau SR, Cronin J, et al. Factors affecting length of stay after posterior spinal fusion for adolescent idiopathic scoliosis. Spine Deform 2020;8(1):51-56. doi: 10.1007/s43390-020-00042-3	2020	LSS	Process mapping	Process	Not described	Process<br>Quality<br>Clinical
McNamara R, Butler A, Baker C, et al. Use of lean principals to improve flow of patients with fractured neck of femur--the HOPE study. Ir Med J 2014;107(3):70-2.	2014	LSS	Waste reduction	Process	Not described	Process
McWilliams A, Schoen M, Krull C, et al. Combining Lean and Applied Research methods to improve rigor and efficiency in acute care outcomes research: A case study. Contemp Clin Trials Commun 2019;14:100322. doi: 10.1016/j.conctc.2019.100322	2019	Lean	VSM	Process	PDCA	Process<br>Clinical
Montella E, Di Cicco MV, Ferraro A, et al. The application of Lean Six Sigma methodology to reduce the risk of healthcare-associated infections in surgery departments. J Eval Clin Pract 2017;23(3):530-39. doi: 10.1111/jep.12662	2017	LSS	SIPOC<br>Cause-and-Effect Diagram	Process<br>Problem Solving	DMAIC	Process<br>Quality
Morales-Contreras MF, Chana-Valero P, Suarez-Barraza MF, et al. Applying Lean in Process Innovation in Healthcare: The Case of Hip Fracture. Int J Environ Res Public Health 2020;17(15) doi: 10.3390/ijerph17155273	2020	Lean	Process mapping<br>Waste reduction	Process<br>Process	Not described	Clinical<br>Process
Murphy C, Mullen E, Hogan K, et al. Streamlining an existing hip fracture patient pathway in an acute tertiary adult Irish hospital to improve patient experience and outcomes. Int J Qual Health Care 2019;31(Supplement_1):45-51. doi: 10.1093/intqhc/mzz093	2019	LSS	SIPOC<br>VSM<br>5 Whys<br>PICK Chart	Process<br>Process<br>Problem Solving<br>Problem Solving	DMAIC	Quality
Niemeijer GC, Flikweert E, Trip A, et al. The usefulness of lean six sigma to the development of a clinical pathway for hip fractures. J Eval Clin Pract 2013;19(5):909-14. doi: 10.1111/j.1365-2753.2012.01875.x	2013	LSS	SIPOC<br>VSM	Process<br>Process	DMAIC	Process
Oetgen ME, Martin BD, Gordish-Dressman H, et al. Effectiveness and Sustainability of a Standardized Care Pathway Developed with Use of Lean Process Mapping for the Treatment of Patients Undergoing Posterior Spinal Fusion for Adolescent Idiopathic Scoliosis. J Bone Joint Surg Am 2018;100(21):1864-70. doi:	2018	LSS	Process mapping	Process	Not described	Clinical<br>Process
Sayeed Z, Anoushiravani A, El-Othmani M, et al. Implementation of a Hip Fracture Care Pathway Using Lean Six Sigma Methodology in a Level I Trauma Center. J Am Acad Orthop Surg 2018;26(24):881-93. doi: 10.5435/JAAOS-D-16-00947	2018	LSS	VSM<br>Kaizen<br>Standardisation of work	Process<br>Problem Solving<br>Process	DMAIC	Quality<br>Process<br>Cost
Schretlen S, Hoefsmit P, Kats S, et al. Reducing surgical cancellations: a successful application of Lean Six Sigma in healthcare. BMJ Open Qual 2021;10(3) doi: 10.1136/bmjoq-2021-001342	2021	LSS	VSM<br>Pareto<br>5 Whys<br>Gemba Walk	Process<br>Problem Solving<br>Problem Solving<br>Problem Solving	DMAIC	Process<br>PREMs
Sethi RK, Pumpian RP, Drolet CE, et al. Utilizing Lean Methodology and Time-Driven Activity-Based Costing Together: An Observational Pilot Study of Hip Replacement Surgery Utilizing a New Method to Study Value-Based Health Care. J Bone Joint Surg Am 2021 doi: 10.2106/JBJS.21.00129	2021	Lean	VSM	Process	Not described	Process<br>Cost
Tekes A, Jackson EM, Ogborn J, et al. How to Reduce Head CT Orders in Children with Hydrocephalus Using the Lean Six Sigma Methodology: Experience at a Major Quaternary Care Academic Children's Center. AJNR Am J Neuroradiol 2016;37(6):990-6. doi: 10.3174/ajnr.A4658	2016	LSS	Kaizen	Problem Solving	DMAIC	Process
Toledo AH, Carroll T, Arnold E, et al. Reducing liver transplant length of stay: a Lean Six Sigma approach. Prog Transplant 2013;23(4):350-64. doi: 10.7182/pit2013226	2013	LSS	Process mapping<br>Cause-and-Effect Diagram<br>5 Whys	Process<br>Problem Solving<br>Problem Solving	DMAIC	Process<br>Quality
Tzadok B, Ben Tov O, Vaispapir V, et al. Lean six sigma and stroke in rural hospital - The case of Baruch Padeh Medical Center. Int J Health Care Qual Assur 2022;ahead-of-print(ahead-of-print) doi: 10.1108/IJHCQA-01-2021-0005	2022	LSS	VSM<br>Cause-and-Effect Diagram	Process<br>Problem Solving	DMAIC	Process<br>Quality
van Vliet EJ, Sermeus W, van Gaalen CM, et al. Efficacy and efficiency of a lean cataract pathway: a comparative study. Qual Saf Health Care 2010;19(6):e13. doi: 10.1136/qshc.2008.028738	2010	Lean	Process mapping	Process	Not described	Process<br>Quality
Verhaert DV, Linz D, Wassink GF, et al. A new efficient and integrated pathway for patient evaluation prior to atrial fibrillation ablation. European Journal of Cardiovascular Nursing 2022	2022	LSS	Process mapping	Process	DMAIC	Process<br>Quality
Wold JFH, Ruiter JH, Cornel JH, et al. A multidisciplinary care pathway for the evaluation of falls and syncope in geriatric patients. Eur Geriatr Med 2015;6(5):487-94. doi: 10.1016/j.eurger.2015.05.007	2015	LSS	Process mapping	Process	DMAIC	Process<br>Clinical
Yousri TA, Khan Z, Chakrabarti D, et al. Lean thinking: can it improve the outcome of fracture neck of femur patients in a district general hospital? Injury 2011;42(11):1234-7. doi: 10.1016/j.injury.2010.11.024	2011	Lean	VSM	Process	Not described	Clinical<br>Process
